# MRI for the detection of small malignant renal masses: a systematic review and meta-analysis

**DOI:** 10.3389/fonc.2023.1194128

**Published:** 2023-10-09

**Authors:** Wenwen Shang, Guohui Hong, Wei Li

**Affiliations:** Department of Medical Imaging, Jiangsu Vocational College of Medicine, Yancheng, China

**Keywords:** MRI, classification, meta-analysis, small renal mass, diagnosis

## Abstract

**Objective:**

We aimed to review the available evidence on the diagnostic performance of magnetic resonance imaging in differentiating malignant from benign small renal masses.

**Methods:**

An electronic literature search of Web of Science, MEDLINE (Ovid and PubMed), Cochrane Library, EMBASE, and Google Scholar was performed to identify relevant articles up to 31 January 2023. We included studies that reported the diagnostic accuracy of using magnetic resonance imaging to differentiate small (≤4 cm) malignant from benign renal masses. The pooled sensitivity, specificity, positive likelihood ratio, negative likelihood ratio, and diagnostic odds ratio were calculated using the bivariate model and the hierarchical summary receiver operating characteristic model. The study quality evaluation was performed with the Quality Assessment of Diagnostic Accuracy Studies-2 tool.

**Results:**

A total of 10 studies with 860 small renal masses (815 patients) were included in the current meta-analysis. The pooled sensitivity and specificity of the studies for the detection of malignant masses were 0.85 (95% CI 0.79-0.90) and 0.83 (95% CI 0.67-0.92), respectively.

**Conclusions:**

MRI had a moderate diagnostic performance in differentiating small malignant renal masses from benign ones. Substantial heterogeneity was observed between studies for both sensitivity and specificity.

## Introduction

In recent decades, with the widespread use of cross-sectional imaging modalities such as ultrasound (US), computed tomography (CT), and magnetic resonance imaging (MRI), the incidence of renal masses has steadily increased (1–3). A higher detection rate of small renal masses (SRMs) leads to an apparent reduction in mortality rates (4); however, whether this depends on the shift towards early-stage disease (which may be easier to treat than high-stage disease) or on improvements in treatment remains to be established (5). Indeed, it is estimated that up to 25% of SRMs ≤3 cm and 30% of SRMs ≤2 cm are benign (6), so precise knowledge of renal cell carcinoma (RCC) subtypes is crucial, as different RCC types lead to different treatment or management strategies. Currently, percutaneous biopsy is considered the gold standard for determining the preoperative histology of an SRM; however, 10–15% of biopsy findings are insufficient for diagnosis. Additionally, for patients who underwent surgical resection, the final pathology results showed that up to 10% of biopsies were misdiagnosed due to tumor heterogeneity (7). Although contrast-enhanced CT (CECT) is the current standard imaging modality, it is difficult to distinguish various RCC subtypes such as ccRCC, papillary RCC (pRCC), and chromophobe RCC (chRCC), which impedes clinicians from making optimal decisions (8, 9).

In recent years, multiparametric MRI (mpMRI) has been intensely studied for histologic subtyping of RCC and in the differentiation of benign from malignant renal masses (10, 11). In mpMRI, T2-weighted images (T2) can be useful in distinguishing between fat-poor angiomyolipomas (AML) and pRCCs, both of which typically exhibit low T2 signal intensity compared to other RCCs (such as ccRCCs and chRCCs) (12). On T1-weighted images (T1) dual-echo chemical shift MRI, ccRCCs and fat-poor AMLs may exhibit a signal decrease in out-phase sequences, which is not commonly observed in oncocytoma. This signal reduction may be seen sporadically in chRCCs or pRCCs, but it tends to be less pronounced compared to ccRCCs and fat-poor AMLs (13). In terms of the apparent diffusion coefficient (ADC) values derived from diffusion-weighted imaging (DWI), AMLs and pRCCs often exhibit low ADC values, whereas for oncocytomas and ccRCCs, these tend to be more heterogeneous and frequently higher. Similar to T2W imaging, chromophobe RCCs typically present with a slightly lower ADC compared to the latter two subtypes (14). Regarding dynamic contrast-enhanced (DCE), ccRCCs and fat-poor AMLs tend to show rapid and intense enhancement during the corticomedullary phase, while the peak of enhancement is slightly delayed in oncocytomas and chRCCs. In all these tumor subtypes, a wash-out pattern of enhancement is observed over time, except for pRCCs, where enhancement tends to increase progressively (10). In this study, we aimed to assess the diagnostic performance of MRI in differentiating small malignant renal masses (cT1a, ≤4 cm) from benign ones.

## Methods

This meta-analysis and systematic review were performed in compliance with the Preferred Reporting Items for Systematic Reviews and Meta-Analysis (PRISMA) statement (15). The primary outcome of our study was the diagnostic accuracy of utilizing MRI for distinguishing between malignant and benign small renal masses.

### Search strategy and selection criteria

An electronic search of PubMed, EMBASE, Cochrane Library, Web of Science, and Google Scholar online databases of scientific publications to identify potentially eligible studies reporting on the relevant topic published up to 31 January 2023 using Medical Subject Headings (MeSH) and restricted to English language. The following terms were used as synonyms for the literature search: ([kidney] OR (renal) OR (nephron)] AND [(cancer) OR (mass*) OR (lesion) OR (carcinoma)] AND ([MRI] OR [MR] OR [magnetic resonance imaging]). An additional search was performed by manually screening the bibliographies of all included studies and reviews to avoid missing potentially eligible studies. Two reviewers (*S.W.W.* and *H.G.H.*) independently assessed the results of the literature search, and disagreements were resolved by discussion until consensus was reached.

### Inclusion and exclusion criteria

Studies were included if they met all of the following criteria: 1) use of MRI for distinguishing malignant small renal masses (≤4 cm) from benign ones; 2) providing sufficient detail to reconstruct 2×2 contingency tables for the determination of diagnostic accuracy; and 3) using histopathological results of biopsy or surgical resection as the reference standard. Studies were excluded that met any of the following criteria: 1) not using MRI but other imaging modalities such as US or CT; 2) with renal masses larger than 4 cm; 3) case reports or case series with too few masses; 4) did not report sufficient data to assess the diagnostic performance; 5) consisted of meta-analyses, guidelines, editorials, reviews, and letters.

### Data extraction and quality assessment

A predefined, standardized form was employed to extract the following data from the included studies: 1) clinical and demographic characteristics, e.g., number of patients and masses, size of masses, age of patients, and male patient/female patient ratio; 2) study characteristics, e.g., first author, study design (prospective or retrospective), year of publication, study site, number of radiologists and their experience, analysis (per person or per lesion), whether blinded to pathological results, and reference standard; 3) technical characteristics, e.g., MRI field strength, sequences, and cutoff values. The Quality Assessment of Diagnostic Accuracy Studies–2 was used to assess the quality of the included studies (16), according to which studies were evaluated in four domains: patient selection domain, index test method domain, reference standard domain, and flow and timing domain. Each study was classified as low, unclear, or high risk of bias for these four domains. Two reviewers (*S.W.W.* and *H.G.H.*) conducted the data extraction and quality assessment independently, and discrepancies were resolved by discussion with the third reviewer (*L.W.*).

### Data synthesis and statistical analysis

In this meta-analysis, we used the bivariate model and the hierarchical summary receiver operating characteristic (HSROC) model to summarize the estimates of sensitivity, specificity, likelihood ratio (LR+), negative likelihood ratio (LR−), and diagnostic odds ratio (DOR) along with their 95% confidence intervals (CIs) (17, 18). In addition, the forest plots and HSROC curves were constructed to graphically demonstrate the results. The Cochran *Q* and Higgins *I*^2^ statistics were used to measure the degree of heterogeneity between studies: for *I*^2^ between 0% and 40%, not important; for *I*^2^ between 30% and 60%, moderate; for *I*^2^ between 50% and 90%, substantial; for *I*^2^ between 75% and 100%, considerable (19). To investigate heterogeneity between studies, meta-regression analyses were performed with the following covariates: magnetic field strength (1.5 T vs. 3.0 T), mass number (<80 vs. ≥80), malignancy rate (<0.6 vs. ≥0.6), analysis (per person vs. per lesion), and year of publication (<2017 vs. ≥2017). All analyses were performed with STATA 16.0 (StataCorp, Texas, USA), with a *P <*0.05 indicating statistical significance.

## Results

### Literature search and data extraction

**Figure 1:**
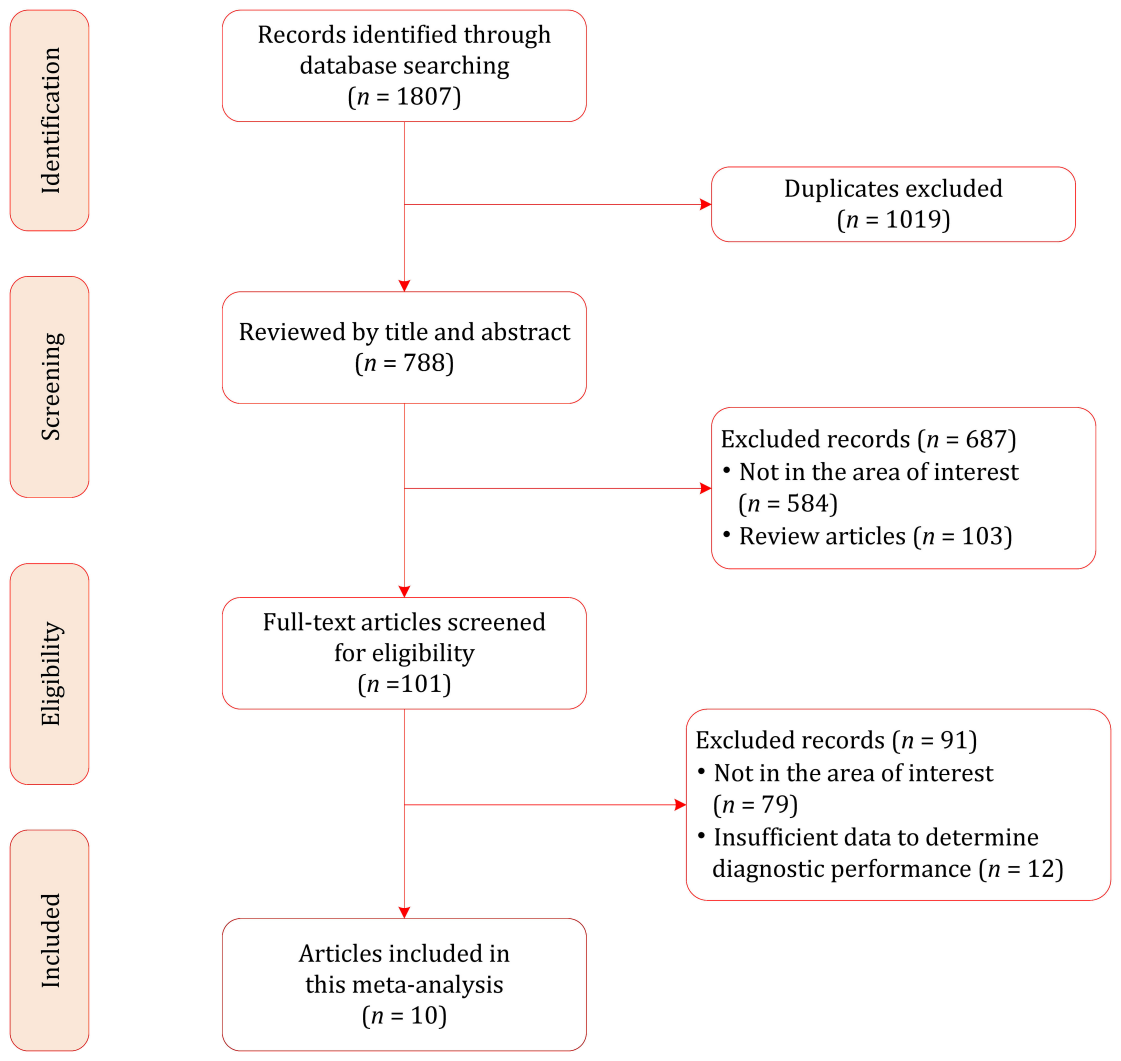
Study selection process for this systematic review and meta-analysis.

Our literature search strategy yielded an initial total of 1807 records, of which 1019 were removed because of duplicates. After screening the titles and abstracts, 687 records were excluded. The full text of the remaining 109 records was reviewed, and 91 were excluded for the following reasons: insufficient data to determine diagnostic performance (*n*=12), not in the area of interest (*n*=79). Finally, a total of 10 studies involving 815 patients (with 860 renal masses) were included in this meta-analysis (12, 20–28); the flow chart of the literature selection process is shown in **Figure 1**.

### Characteristics of included studies

The detailed demographic characteristics are provided in **Table 1**. The sample size of the population ranged from 39 to 158 participants (46-170 renal masses), with a mean age of 50-66 years, and an average tumor size of 19-33 mm. In seven studies, the MRI images were interpreted by two readers; in two studies, the images were interpreted by three readers, whereas in one study, the number of readers was not reported. The level of experience of the radiologists was heterogeneous, ranging from 2 to 19 years. With regard to magnetic field strength, nearly all studies reported that the images were acquired with 1.5 T and 3.0 T scanners or both. As for the MRI protocol, only one study used all sequences of T1, T2, DCE, and DWI; our studies used three sequences (T1, T2, and DCE); and four studies used T1, T2, and DWI. Nine studies used the pathological results of surgical resection as the reference standard, while the remaining study used the pathological results of percutaneous biopsy combined with 5 years of follow-up as the reference standard. **Table 2** provides detailed study characteristics.

**Table 1:** Demographic characteristics of the included studies.

First Author	Country	Year	Patient Number	Masses	Gender	Age (year, mean ± SD/median)	Tumor Size (cm, mean ± SD/median)	Subtypes
**Dunn**	Canada	2022	102	108	67/53	56.9 ± 12.8	3.0 ± 1.3	ccRCC/pRCC/chRCC/Onco/AML
**Kim**	Korea	2016	68	68	47/21	63.1	NA	ccRCC/pRCC/chRCC/Onco/AML/Others
**Li**	China	2018	92	92	59/33	52/49^a^	3.0	ccRCC/pRCC/chRCC/Onco/AML
**Ludwig**	USA	2020	95	95	51/44	61 ± 14	2.7 ± 0.9/1.5 ± 0.7^b^	ccRCC/pRCC/chRCC/Onco/Cyst
**Mytsyk**	Ukraine	2017	158	170	97/61	53.6 ± 14.9	3.31 ± 0.69	ccRCC/Others
**Park**	Korea	2018	50	50	23/27	51.0 ± 13.0/ 49.9 ± 10.3^c^	2.3 ± 0.7/ 1.9 ± 0.6	ccRCC/pRCC/chRCC/Others
**Ponhold**	Austria	2015	39	46	NA	66.2 ± 11.8	NA	NA
**Sasiwimonphan**	Thailand	2012	111	119	69/42	59.7	2.4/2.1^c^	ccRCC/pRCC/chRCC/AML/Others
**Willatt**	USA	2014	51	63	24/27	58/62^a^	< 2	NA
**Zhang**	China	2015	49	49	26/23	21-70	3.2 ± 1.2	ccRCC/Others

AML, angiomyolipoma; ccRCC, clear cell renal cell carcinoma; chRCC, chromophobe renal cell carcinoma; NA, not available; Onco, oncocytoma; pRCC, papillary renal cell carcinoma; SD, standard deviation.

a Man/Woman.

b Malignant/Benign.

c RCC/AML.

**Table 2:** Study characteristics of the included studies.

First Author	Study Design	Period	No of Readers	Experience (Years)	Magnet Field Strength	Blinded	MRI Sequence	Assessment	Cutoff Value	Reference
**Dunn**	Retrospective	2013.01-2018.02	3	7-12	1.5 T	Yes	T1/T2/DCE	ccLS	≥4	Surgical pathology
**Kim**	Retrospective	2008.02-2013.02	2	NA	3.0 T	Yes	T1/T2/DCE/DWI	DCE	NA	Surgical pathology
**Li**	Retrospective	2014.07-2016.05	2	3/12	3.0 T	Yes	T1/T2/DWI	ADC value	90th percentile	Surgical pathology
**Ludwig**	Retrospective	2010.06-2018.07	2	3/6	1.5 T/3.0 T	Yes	T1/T2/DWI	ADC ratio	0.89	Biopsy+5 years follow-up /Surgical pathology
**Mytsyk**	Retrospective	2013–2017	NA	NA	1.5 T	NA	T1/T2/DWI	ADC value	1.75×10^-3^ mm^2^/s	Surgical pathology
**Park**	Retrospective	2009.01-2016.12	2	5/14	1.5 T	Yes	T1/T2/DCE	T2 ratio	0.783	Surgical pathology
**Ponhold**	Retrospective	NA	2	5/8	3.0 T	Yes	T2/DWI	ADC value	0.99×10^-3^ mm^2^/s	Surgical pathology
**Sasiwimonphan**	Retrospective	2003.01-2011.01	2	2/19	1.5 T/3.0 T	Yes	T1/T2/DCE	T2 ratio	0.9	Surgical pathology
**Willatt**	Retrospective	2001.01-2007.12	2	3/10	1.5 T	Yes	T1/T2/DCE	T1/T2/DCE	NA	Surgical pathology
**Zhang**	Prospective	2011.03-2014.04	3	NA	3.0 T	Yes*	T1/T2/DWI	ADC value	1.36×10^-3^ mm^2^/s	Surgical pathology

ADC, apparent diffusion coefficient; ccLS, clear cell likelihood score; DCE, dynamic contrast enhanced; DWI, diffusion weighted imaging; T1, T1 weighted imaging; T2, T2 weighted imaging; NA, not available.

### Quality assessment

**Figure 2:**
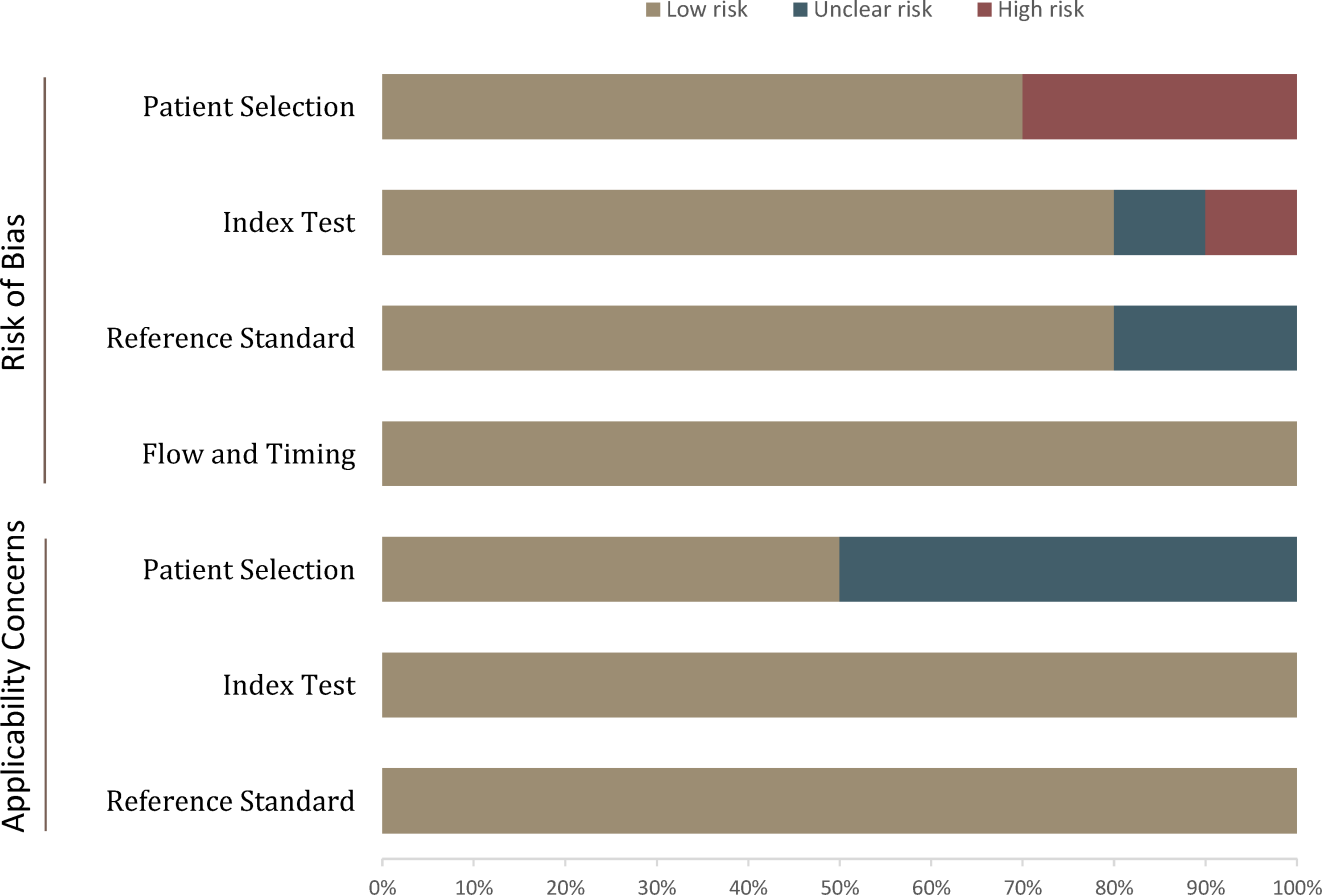
Grouped bar charts show the risk of bias and concerns about the applicability of the included studies.

The overall quality assessment of the included studies was high. Concerning patient selection, three studies were classified as having a high risk of bias, mainly because lesions smaller than 1 cm were excluded (20, 26, 28). For application concerns, five studies were classified as having an unclear risk of bias because the malignancy rate was too high (20–22, 25, 27). For the index domain, two studies did not explicitly report blinding or had known partial patient information and were therefore classified as unclear or high risk of bias (27, 28). With respect to the flow and timing domains, all studies were assessed as low risk of bias; the detailed quality assessment is shown in **Figure 2** and **Table S1**.

### Diagnostic performance of MRI for SRM

**Figure 3:**
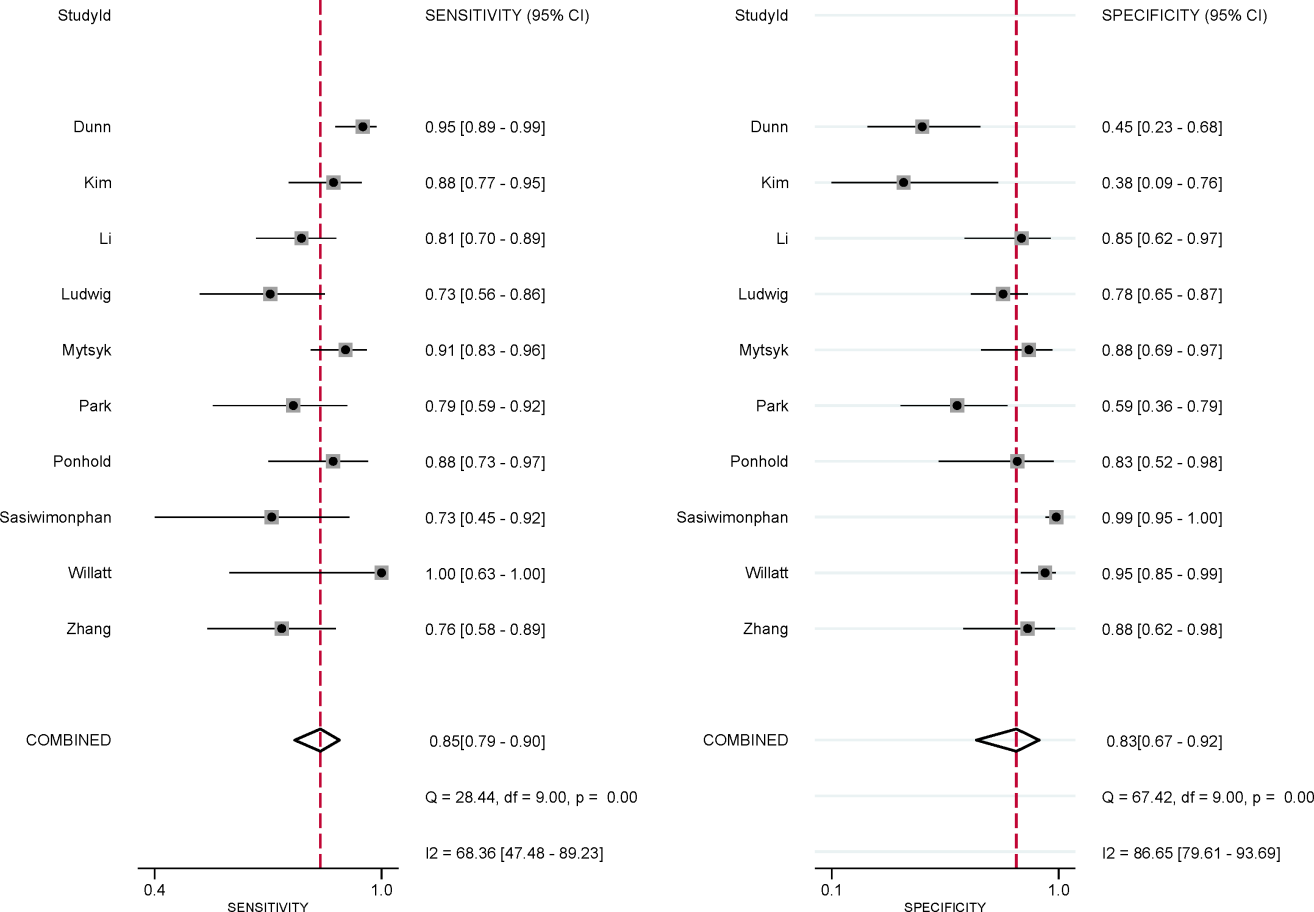
Coupled forest plot of pooled sensitivity and specificity for studies on the detection of malignant masses. Numbers are pooled estimates with 95% CIs in parentheses. Corresponding heterogeneity statistics are provided in the lower right corner. Horizontal lines indicate 95% confidence intervals.

**Figure 4:**
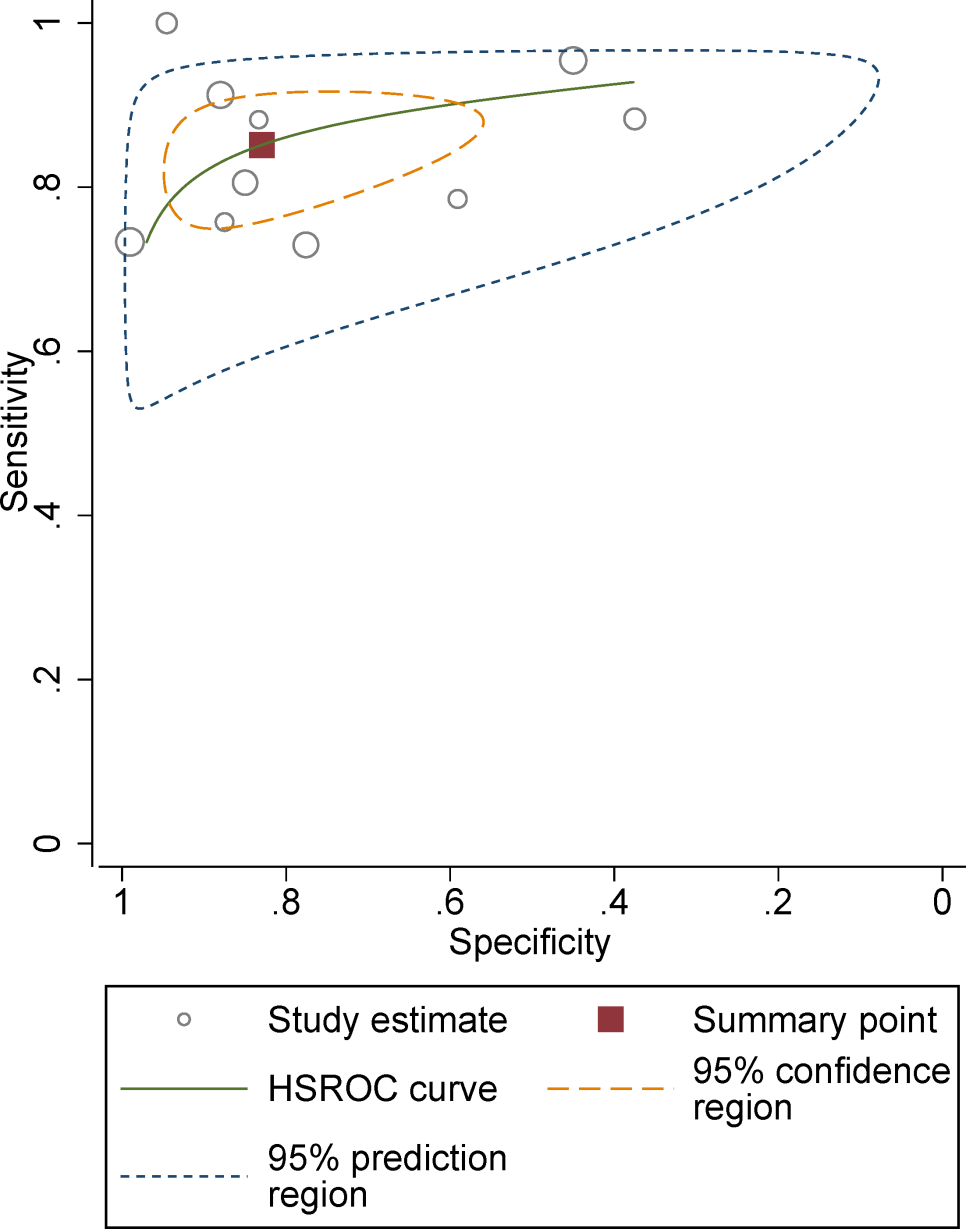
Hierarchical summary receiver operating characteristic plots with summary point and 95% confidence interval for studies on differentiation of malignant masses.

The pooled summary estimates of sensitivity and specificity were 0.85 (95% CI 0.79-0.90) and 0.83 (95% CI 0.67-0.92), respectively, with a calculated area under the HSROC of 0.90 (95% CI 0.87-0.92). For the individual study, the sensitivity and specificity were 0.73-1.00 and 0.38-0.99, respectively; the corresponding forest plots are shown in **Figure 3**. The pooled LR+, LR−, and DOR were 5.0 (95% CI 2.5-10.2), 0.18 (95% CI 0.13–0.25), and 28 (95% CI 12-64), respectively. The *Q* test revealed substantial heterogeneity across studies (P<0.05), *I^2^* values suggested substantial heterogeneity in terms of sensitivity (*I^2 =^* 68.3%) and specificity (*I^2 =^* 86.7%). A large difference between the 95% confidence region and the 95% prediction region also indicated substantial heterogeneity (**Figure 4**). To explore the source of heterogeneity, meta-regression analyses were performed on several potential factors; however, we found that none were significantly associated with the heterogeneity, with *P* values ranging from 0.06 to 0.97. Nevertheless, studies using 1.5 T MRI had a higher sensitivity (0.91 vs. 0.83) compared with 3.0 T scanners, even though the difference was not statistically significant (P=0.06); details are presented in **Table S2**.

## Discussion

In this meta-analysis, we summarized the current evidence on the use of MRI for characterizing small renal masses. Based on 10 studies, the pooled sensitivity and specificity for the detection of malignant renal masses were 0.85 and 0.83, respectively. In a recent study assessing bpMRI or mpMRI for solid renal masses, the summary estimate of sensitivity and specificity for distinguishing malignant masses from benign ones was 0.95 and 0.63, respectively (29). The primary difference between our study and theirs was that we focused on small renal masses, i.e., smaller than 4 cm, so most of the works included in the previous meta-analysis were excluded from the current study. In the current meta-analysis, we observed substantial heterogeneity between studies. To identify the causes, we performed meta-regression analyses on several potential variables. However, no variable was found to be significantly associated with heterogeneity. Nevertheless, studies using 1.5 T MRI showed a higher sensitivity than those using 3.0 T (0.91 vs. 0.83), even though the difference was not statistically significant. Other clinical variables, such as imaging protocols (T2, DCE, and DWI), study population, and radiologist experience, were also potential sources of heterogeneity.

T2 MRI has been suggested as the first imaging sequence to be used in the initial assessment of small solid renal masses for potential MRI subtyping. On T2 images, fat-poor AMLs and pRCCs show low signal intensity, which differs significantly from ccRCCs and oncocytomas. Therefore, T2 is highly accurate in separating fat-poor AMLs and papillary RCCs from clear cell RCC and oncocytoma and, when combined with other features can further separate the diagnoses with high accuracy. On DCE images, fat-poor AML usually shows early, strong enhancement with subsequent washout. Although ccRCCs often show a similar pattern of enhancement, the degree of washout tends to be greater in fat-poor AMLs, which is useful for differentiating fat-poor AMLs and ccRCCs from two other RCC subtypes of pRCC and chRCC, which show less enhancement during the corticomedullary phase. Recently, more studies have investigated the role of ADC values (including mean ADC value and ratio) in differentiating subtypes of RCC. Preliminary studies reported that pRCCs have lower ADC values as compared to other renal tumors such as oncocytomas or ccRCCs; however, different MRI manufacturers lead to different *b* values, making quantitative assessment difficult to reproduce; moreover, the optimal cutoff values reported in the studies vary widely.

Currently, both the American Urologic Association and the American Society of Clinical Oncology recommend active surveillance as an initial management strategy for incidental small renal masses, based on the rationale that only 20% of cT1a RCCs are high-grade and associated with disease progression and metastasis, whereas most are indolent. Nevertheless, surveillance of clear cells and potentially other high-grade small RCCs may occasionally yield unfavorable outcomes; thus, differentiating between RCCs and benign ≤4 cm solid renal masses is highly desirable to optimize treatment. Pretreatment histological diagnosis of renal masses can be achieved by renal mass biopsy; however, the potential for non-diagnostic yield and sampling error has restricted its widespread adoption. Furthermore, renal mass biopsy is not feasible for all patients as it is still invasive (30, 31). Therefore, the use of noninvasive imaging modalities to distinguish malignant renal masses from benign ones is clinically desirable (32). In general, active surveillance is more favorable for elderly patients with comorbid conditions or a limited life expectancy; for others, choosing active surveillance must be based on a risk/balanced benefit.

Our study has several limitations. First, most of the included articles were retrospective in their study design, which led to a high risk of bias in the area of patient selection. However, because only one study was prospective, it was not possible to obtain summary estimates from prospective studies. Second, substantial heterogeneity was observed between studies, which may potentially limit the generalizability of the results. Although meta-regression analyses were performed on several potential factors to explore the sources, this only accounted for part of the heterogeneity.

## Conclusion

The use of MRI for detecting malignant small renal masses and distinguishing ccRCCs from other subtypes of cancer yielded moderate diagnostic performance. Substantial heterogeneity among studies was noted in terms of sensitivity and specificity. Awareness of these diagnostic performance results will be helpful as MRI is increasingly implemented into clinical practice for the assessment of renal masses.

## Data availability statement

The original contributions presented in the study are included in the article/**Supplementary Material**. Further inquiries can be directed to the corresponding author.

## Author contributions

All authors listed have made a substantial, direct, and intellectual contribution to the work and approved it for publication.
