# Supplementary material for: MRI for the detection of small malignant renal masses: a systematic review and meta-analysis
Source: Front Oncol. 2023 Oct 9;13:1194128. doi: 10.3389/fonc.2023.1194128 (PMC10591109; doi:10.3389/fonc.2023.1194128)
Supplement: Supplementary file 1 [file Table_1.docx]

**Table S1.** Quality assessment according to QUADAS-2

| **First Author** | **Risk of Bias** | | | | **Concern of Applicability** | | | **Reason for High or Unclear Assessments** |
| --- | --- | --- | --- | --- | --- | --- | --- | --- |
|  | **Patient Selection** | **Index**  **Test** | **Reference Standard** | **Flow and Timing** | **Patient Selection** | **Index Test** | **Reference Standard** |  |
| **Dunn** | High | Low | Unclear | Low | Unclear | Low | Low | Lesions of <1 cm in size were excluded  biopsy  Too high malignant rate |
| **Kim** | Low | Low | Low | Low | Unclear | Low | Low | Too high malignant rate |
| **Li** | Low | Low | Low | Low | Unclear | Low | Low | Too high malignant rate |
| **Ludwig** | Low | Low | Unclear | Low | Low | Low | Low | biopsy |
| **Mytsyk** | Low | Low | Low | Low | Low | Low | Low |  |
| **Park** | High | Unclear | Low | Low | Low | Low | Low | Blinding: NA  Lesions of <1 cm in size were excluded |
| **Ponhold** | Low | Low | Low | Low | Unclear | Low | Low | Too high malignant rate |
| **Sasiwimonphan** | Low | Low | Low | Low | Low | Low | Low |  |
| **Willatt** | High | Low | Low | Low | Low | Low | Low | Indeterminate on prior US or CT, and not consecutive |
| **Zhang** | Low | High | Low | Low | Unclear | Low | Low | Blinding: knowing partial information.  Too high malignant rate |
