# Supplementary material for: MRI for the detection of small malignant renal masses: a systematic review and meta-analysis
Source: Front Oncol. 2023 Oct 9;13:1194128. doi: 10.3389/fonc.2023.1194128 (PMC10591109; doi:10.3389/fonc.2023.1194128)
Supplement: Supplementary file 2 [file Table_2.docx]

**Table S2. Meta-Regression**

| **Covariate** | | **Sensitivity** | **Specificity** | ***P*** Value |
| --- | --- | --- | --- | --- |
| Magnet field strength | 1.5 | 0.91/0.82-0.95 | 0.78/0.50-0.91 | 0.06 |
|  | 3.0 | 0.83/0.78-0.88 | 0.78/0.58-0.92 | 0.97 |
| Patient No. | <80 | 0.84/0.78-0.91 | 0.79/0.56-0.93 | 0.84 |
|  | >80 | 0.85/0.74-0.91 | 0.86/0.62-0.95 | 0.57 |
| Malignant rate | <0.6 | 0.83/0.72-0.90 | 0.89/0.71-0.96 | 0.42 |
|  | >0.6 | 0.87/0.79-0.92 | 0.73/0.51-0.89 | 0.17 |
| Publication  Year | <2017 | 0.84/0.75-0.91 | 0.90/0.67-0.97 | 0.66 |
|  | ≥2017 | 0.86/0.77-0.92 | 0.73/0.58-0.89 | 0.15 |
| Analysis | Per person | 0.80/0.71-0.88 | 0.68/0.49-0.88 | 0.21 |
|  | Per lesion | 0.88/0.79-0.92 | 0.90/0.72-0.96 | 0.10 |
